# Physicochemical profiles of mixed ruminal microbes in response to surface tension and specific surface area

**DOI:** 10.3389/fvets.2024.1514952

**Published:** 2025-01-06

**Authors:** Yong Liu, Junrui Liao, Shaoxun Tang, Chuanshe Zhou, Zhiliang Tan, Abdelfattah Z. M. Salem

**Affiliations:** ^1^CAS Key Laboratory for Agro-Ecological Processes in Subtropical Region, National Engineering Laboratory for Pollution Control and Waste Utilization in Livestock and Poultry Production, Hunan Provincial Key Laboratory of Animal Nutrition and Physiology and Metabolism, Institute of Subtropical Agriculture, the Chinese Academy of Sciences, Changsha, Hunan, China; ^2^College of Advanced Agricultural Sciences, University of Chinese Academy of Science, Beijing, China; ^3^Facultad de Medicina Veterinaria y Zootecnia, Universidad Autónoma del Estado de México, Toluca, Mexico

**Keywords:** physicochemical properties, rumen microbes, specific surface area, surface tension, physical properties

## Abstract

**Introduction:**

In ruminants, a symbiotic rumen microbiota is responsible for supporting the digestion of dietary fiber and contributes to health traits closely associated with meat and milk quality. A holistic view of the physicochemical profiles of mixed rumen microbiota (MRM) is not well-illustrated.

**Methods:**

The experiment was performed with a 3 × 4 factorial arrangement of the specific surface area (SSA: 3.37, 3.73, and 4.44 m^2^/g) of NDF extracted from rice straw and the surface tension (ST: 54, 46, 43, and 36 dyn/cm) of a fermented medium in a fermentation time series of 6, 12, 24, 48 h with three experimental units. Here, we used three rumen-fistulated adult Liuyang black goats as the rumen liquid donors for this experiment.

**Results:**

It was found that increasing SSA decreased the average acetate/propionate ratio (A/P, *p* < 0.05) and increased the molarity of propionate (*p* < 0.05). Increasing ST decreased total volatile fatty acid (tVFA) concentration (*p* < 0.01). Greater SSA increased (*p* < 0.01) MRM hydrophobicity, whereas increasing ST increased MRM cell membrane permeability (*p* < 0.01). The neutral detergent fiber digestibility (NDFD, *r* = 0.937) and tVFA (*r* = 0.809) were positively correlated with the membrane permeability of MRM.

**Discussion:**

The surface tension of the artificial medium and substrate-specific surface area had a significant influence on MRM's fermentation profiles, hydrophobicity, and permeability. The results suggest that physical environmental properties are key in regulating rumen fermentation function and homeostasis in the gastrointestinal tract ecosystem.

## Implications

Enzyme activity and microbes' adhesion to the substrate are directly related to the physical characteristics of the substrate and rumen fluid. We measured the *in vitro* fermentation of neutral detergent fiber and the surface physical properties of microbes under the different specific surface areas of the neutral detergent fiber and surface tension of the culture medium. This findings indicate that rumen fermentation and animal production can be improved by modifying the physical characteristics of the substrate and rumen fluid.

## Introduction

For ruminants, the rumen microbiota is responsible for degrading indigestible fibers into energy and nutrients (1). The ability of the microbiota to digest fiber is enhanced when they transform from planktonic to irreversible adhesion model (2). Microbial biofilms are the prerequisite for fiber digestion and microbial adhesion (3). Microbial biofilms are multicellular microbial communities formed after planktonic cells adhere to solid surfaces (4). The transition from a free-living planktonic lifestyle to a sessile, attached state, forming biofilms, is a multifactorial process governed by biological, chemical, and physical properties of the environment, surface, and bacterial cell (5). Bacterial biofilm is a matrix of extracellular polymeric substances (EPS) (6) that provide mechanical stability and protection against environmental adversities (7). The biofilm is influenced by the availability of environmental nutrients and some nucleotide second messengers, such as cyclic adenosine monophosphate (cAMP) and cyclic di-guanosine monophosphate (c-di-GMP) through cAMP receptor protein (CRP) signaling (8).

The initial bacterial adhesion process is influenced by the properties of the material surface, including surface roughness, topography, wettability, and stiffness (7, 9, 10). The structured channels within the biofilm (3) facilitate the exchange of nutrients between the embedded microbes and the external environments, which contribute to microbial colonization (11) and quorum sensing (12). Specific surface area (SSA) is a crucial parameter in quantifying interactive functions at liquid-solid/gas interfaces, especially in the case of adsorption, heterogeneous catalysis (13), and reaction at the surface of materials (14, 15). Feed particles with greater SSA provide more adsorption sites, resulting in higher fiber digestion efficiency (7). Bacterial attachment and biofilm formation on substrate surfaces highly depend on the available surface area (16, 17). However, comprehensive data linking SSA with microbial adhesion and biofilm formation are still lacking, representing a significant knowledge gap.

Surface tension (ST) of the media also plays a critical role in microbial attachment abilities and the adhesion process, such as its superhydrophobic or super-hydrophilic properties (7, 18). This study is novel in its approach to systematically evaluating the combined effects of SSA and ST on the physical properties of the rumen microbiota and fermentation profiles, an area that has not been extensively explored. The interaction between these factors and their influence on microbial cell membrane permeability, hydrophobicity, and fermentation efficiency remain poorly understood, despite their potential to optimize microbial activity and feed digestibility.

From an economic perspective, improving the efficiency of rumen fermentation is crucial for sustainable livestock production (19). Feed costs account for up to 70% of total expenses in ruminant farming (20). Enhancing fiber digestibility through physicochemical modifications to feed properties offers a cost-effective strategy to maximize feed utilization and minimize waste (21). Additionally, optimizing fermentation conditions, such as reducing ST, can further improve microbial efficiency, leading to higher productivity and lower feed costs (22, 23). These strategies are particularly valuable in the context of increasing global demand for livestock products and the need for sustainable agricultural practices.

This study aims to address these gaps by employing a factorial experimental design to evaluate how varying SSA and ST levels influence the physical and chemical properties of rumen microbes, as well as their fermentation capabilities. By integrating these physicochemical parameters, our findings offer actionable strategies for improving feed efficiency, livestock productivity, and environmental sustainability (24, 25). Additionally, the strong correlations observed between microbial cell membrane permeability and key fermentation metrics, such as fiber digestibility and volatile fatty acid (VFA) production, provide a mechanistic understanding that could guide future advancements in ruminant nutrition and biotechnology.

An in-depth understanding of the physical properties of the environmental surface on fermentation kinetics and physicochemical properties of the rumen microbiota could be conducive to regulating the fiber degradation process. Therefore, this study was conducted to investigate the effects of ST of the fermentation inoculum and SSA of the substrate on the physical properties of the rumen microbes and the consequences of fiber degradation.

## Materials and methods

### Experimental design

The experiment consisted of a series of *in vitro* batch cultures performed as a completely randomized experimental design with 12 treatments in a 3 × 4 factorial arrangement, each comprising three individual runs. The present work used three SSAs of neutral detergent fiber (NDF) (3.37, 3.73, and 4.44 m^2^/g) and four STs (36, 43, 46, and 54 dyn/cm) of the incubation medium. Another run was performed on separate days using the same SSA preparations. The new ST was similar to the previous run and was prepared with fresh rumen inoculum collected from three experimental goats at a similar ratio. The experiment was approved by the Animal Care Committee of the Institute of Subtropical Agriculture, Chinese Academy of Sciences, Changsha, Hunan (No. ISA-2012-018).

### Inoculum donator and *in vitro* fermentation

In the present work, animal welfare and management strategies of rumen inoculum donors were described in our previous work (23, 26). Briefly, three rumen-fistulated adult black Liuyang goats of similar age, weight, and good health status were used to provide equal rumen fluids of about 150 ml rumen liquid of each donor for *in vitro* fermentation to reveal the physical characteristics of ST and SSA. The *in vitro* fermentation procedure and chemical compositions of the buffer medium were explained in our previous publications (15, 27). About 50 mL of fermentation medium at a ratio of 1:2 (v/v, rumen fluid: buffer medium) with 4 ST properties, combined with 500 ± 50 mg neutral detergent fiber (NDF) of rice straw (3 SSA levels) at 39°C under continuous flushing with CO_2_, was performed in a time series of 6, 12, 24, and 48 h to collect fermented microbial samples. The fermented samples were used to determine volatile fatty acid (VFA) concentrations and physical properties (zeta potential, also called electrokinetic potential, hydrophobicity, and cell membrane permeability) of the microbiota in the rumen. The diet for goats consisted of a concentrate (corn 47%, soybean meal 24%, wheat bran 22%, salt 0.77%, limestone powder 2.23%, and premix 4%) and forage (corn stover) in a ratio of 40:60. The goats were provided free access to water and were fed twice daily at 08:00 and 18:00, receiving 200 g of concentrate and 300 g of forage per day.

### Specific surface area and surface tension

The NDF resource was derived from rice straw, while the surface tension system was constructed as described in our previous work (23). Briefly, 3 SSAs (3.37, 3.73, and 4.44 m^2^/g) of NDF were ground, sieved through a mill with different sieve pore sizes, and measured using a surface area analyzer (Quadrasorb-*SI*, Quantachrome Inc. Florida, CA, USA). Four STs (36, 43, 46, and 54 dynes/cm) of inoculum medium were prepared by adding alkyl polyglucoside (APG, 28.7 dynes/cm) and immediately measured using a model K100 tensiometer (KRÜSS GmbH, Hamburg, Germany).

### Analysis of physical properties of microbes

The mixed fermentation samples, consisting of a mixture of fermentation substrate and microbes, were collected at a fermentation time series of 6, 12, 24, and 48 h. The mixed fermentation samples of 2, 3, and 2 mL of supernatant were collected by centrifugation (5,000 × g at 4°C for 10 min to remove the substrate) to analyze the zeta potential (ξ), cell membrane permeability, and hydrophobicity of the mixed rumen microbes, respectively. To measure the mixed rumen microbiota's physical properties, we followed our previous work's detailed descriptions (15, 23, 28).

#### Zeta potential (ξ)

The microbial pellet was prepared by rinsing and resuspending the collected residue in 5 mL of 1 mM KNO_3_ (pH 6.6) as described by Pelletier et al. (29) and determined using a zeta potential analyzer (Brookhaven Instruments Corp., Holtsville, NY, USA) equipped with a He-Ne laser (658.0 nm) as the light source in a high-precision system (30 cycles) at 39°C.

#### Permeability of cell membrane

Three milliliter of supernatant was mixed with 1 mL of 100 mg/L fluorescein isothiocyanate-dextran (FITC-dextran, Sigma, molecular weight ≈ 38 kDa) solution and incubated for 1 h at 37°C and then centrifuged to collect 2 mL of sub-supernatant for fluorescence intensity determination (15, 30). The blank negative control was prepared using 3 mL supernatant and 1 mL distilled water.

#### Hydrophobicity of the cell surface

The microbiota pellet from each fermented flask was centrifuged, collected, rinsed, homogenized, and resuspended in 6 mL of 0.1 M KNO_3_ phosphate-buffered saline (PBS) solution (pH 6.6) as described in previous reports (23, 31). The hydrophobicity and permeability of the microbial cell membrane were calculated according to Equations 1, 2 (23), respectively.


(1) $$\begin{array}{l} Hydrophobicity\left(\%\right)=\frac{H_{a0}-H_{a1}}{H_{a0}}\times 100\% \end{array}$$

(2) $$\begin{array}{l} Permeability\left(\%\right)=\frac{P_{a0}-P_{a1}}{P_{a0}}\times 100\% \end{array}$$

Hydrophobicity was defined as the difference between the percentage of cells retained by the hydrocarbon and aqueous layers. *H*_*a*0_ represents the absorbance value of optical density (OD) value at 400 nm, OD400, before the addition of hexadecane, and *H*_*a*1_ represents the absorbance value of OD400 after hexadecane addition. *P*_*a*0_ represents the absorbance value of 25 mg/L FITC-dextran rumen microbe-free liquid, and *P*__*a*_1_ represents the incubated microbe-free liquid absorbance value.

### Chemical analysis

The concentration of VFA in the fermentation liquid was determined according to our previous report (28). Two milliliter of fermented liquid was centrifuged, collected, immediately mixed, and homogenized with 0.15 mL metaphosphoric acid (conc. 25%). The sub-supernatant was then centrifuged and collected for analysis of VFA concentrations at incubation time series of 6, 12, 24, and 48 h by gas chromatography (HP5890, Agilent 5890; Agilent Technologies Co. Ltd, USA).

### Statistical analysis

Statistical analysis of a completely randomized experimental design was performed using the MIXED procedure of SAS software (32). For VFA and surface physical variables, the SSA, ST, time series, and interactions were included in the model as fixed effects, while the run was used as a random effect and incubation time as a repeated effect. The SLICE statement of SAS software was used to detect differences between means at specific SSA levels, and the pairwise difference (PDIFF) statement of SAS software was used to compare ST effects within each SSA level. Pearson correlation analysis was performed using the *Proc Corr* procedure of SAS (32), and *r* > 0.5 was considered biologically significant. Least-squares means are given throughout the text, and significance was reported as *P* ≤ 0.05.

## Results

### Volatile fatty acids

**Figure 1:**
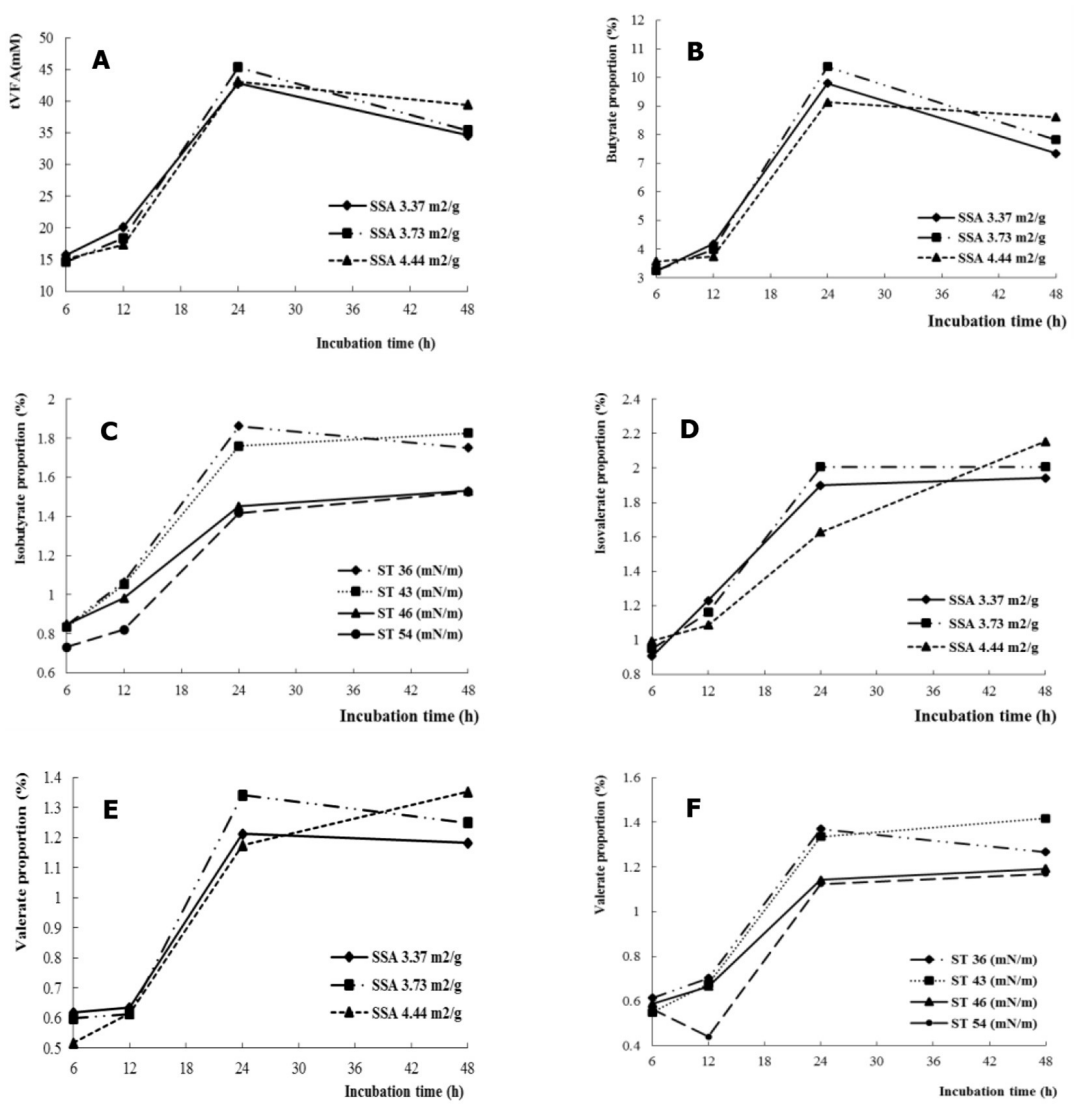
The SSA or ST interacted with fermentation time on VFA profiles, tVFA **(A)**, butyrate **(B)**, isobutyrate **(C)**, isovalerate **(D)**, and valerate **(E, F)**.

The propionate was affected by SSA, which increased with increasing SSA (*P* < 0.05) (Table 1). In addition, the ratio of acetate to propionate decreased with increasing SSA (*P* < 0.05). The tVFA and isovalerate decreased (*P* < 0.01), but propionate increased (*P* < 0.01) with increasing ST. Interactive effects for NDFD, tVFA, and individual VFA were not observed between SSA and ST. A similar interaction for tVFA (Figure 1A), butyrate (Figure 1B), and isobutyrate (Figure 1C) was observed between time and SSA. Valerate and isovalerate increased with increasing time (SSA = 4.44 m^2^/g), whereas they decreased after a fermentation time longer than 24 h (SSA = 3.37 and 3.73 m^2^/g, respectively) (SSA × time interaction, *P* < 0.05; Figures 1D, E). In addition, isobutyrate and valerate increased with fermentation time as a function of ST of the fermentation liquid (ST × time interaction, *P* < 0.05 for isobutyrate, Figure 1D, and ST × time interaction, *P* < 0.01 for isobutyrate, Figure 1F).

**Table 1:** Combinatorial interactions of SSA of NDF, ST, and incubation time on VFA profiles.

**Item^1^**	**NDFD (%)^2^**	**tVFA (mM)^3^**	**Profiles of volatile fatty acids (mol/100 mol)**						**A:P^4^**
			**Acetate**	**Propionate**	**Butyrate**	**Isobutyrate**	**Isovalerate**	**Valerate**	
**SSA (m** ^2^ **/g)**									
3.37	22.6 ^a^	28.3	41.0	24.1 ^b^	21.4	4.63	5.50	3.35	1.72^a^
3.73	21.9^b^	28.4	38.4	25.3 ^ab^	22.3	4.89	5.74	3.50	1.53^ab^
4.44	22.3^ab^	28.7	39.3	25.9 ^a^	22.2	4.79	5.56	3.30	1.51^b^
SEM^a^	0.16	0.78	0.61	0.27	0.23	0.079	0.085	0.053	0.035
**ST (dynes/cm)**									
36	17.6^c^	29.6^ab^	39.3	24.3^b^	22.1	4.82	6.05 ^a^	3.43	1.64
43	24.1^a^	30.7^a^	39.3	24.5^b^	22.3	4.83	5.64 ^ab^	3.37	1.62
46	24.0^a^	27.3^b^	39.3	25.1^b^	21.9	4.75	5.46 ^b^	3.44	1.59
54	23.2^b^	26.4^b^	38.7	26.4^a^	21.6	4.68	5.25 ^b^	3.30	1.49
SEM^b^	0.19	0.86	0.74	0.31	0.29	0.091	0.102	0.065	0.042
**Significance of effects**									
SSA	^*^	NS	NS	^*^	NS	NS	NS	NS	^*^
ST	^***^	^***^	NS	^**^	NS	NS	^**^	NS	NS
SSA ^*^ ST	NS	NS	NS	NS	NS	NS	NS	NS	NS
Time	^***^	^***^	^***^	^**^	NS	^***^	^***^	^***^	^**^
SSA ^*^ time	^***^	^*^	NS	NS	^*^	NS	^*^	^*^	NS
ST ^*^ time	^***^	NS	NS	NS	NS	^*^	NS	^**^	NS
SSA ^*^ ST ^*^ time	^***^	NS	NS	NS	NS	NS	NS	^**^	NS

^1^SSA, specific surface area; ST, surface tension; SEM^a^, standard error of least squares means for SSA; SEM, standard error of least squares means for ST; SSA ^*^ ST, the interaction between SSA and ST; Time, incubation time; SSA ^*^ time, the interaction between specific surface area and incubation time; ST ^*^ time, the interaction between surface tension and time; SSA ^*^ ST ^*^ time, interaction among SSA, ST, and time.

^2^NDFD, neutral detergent fiber disappearance.

^3^tVFA, total volatile fatty acid; NS means no significance; ^*^, ^**^ and ^***^ mean *P* < 0.05, *P* < 0.01, and *P* < 0.001, respectively.

^4^A:P, the ratio of acetate to propionate.

^a, b, c^Means with different superscripts differed (*P* < 0.05).

### Physical properties of the rumen microbiota

**Figure 2:**
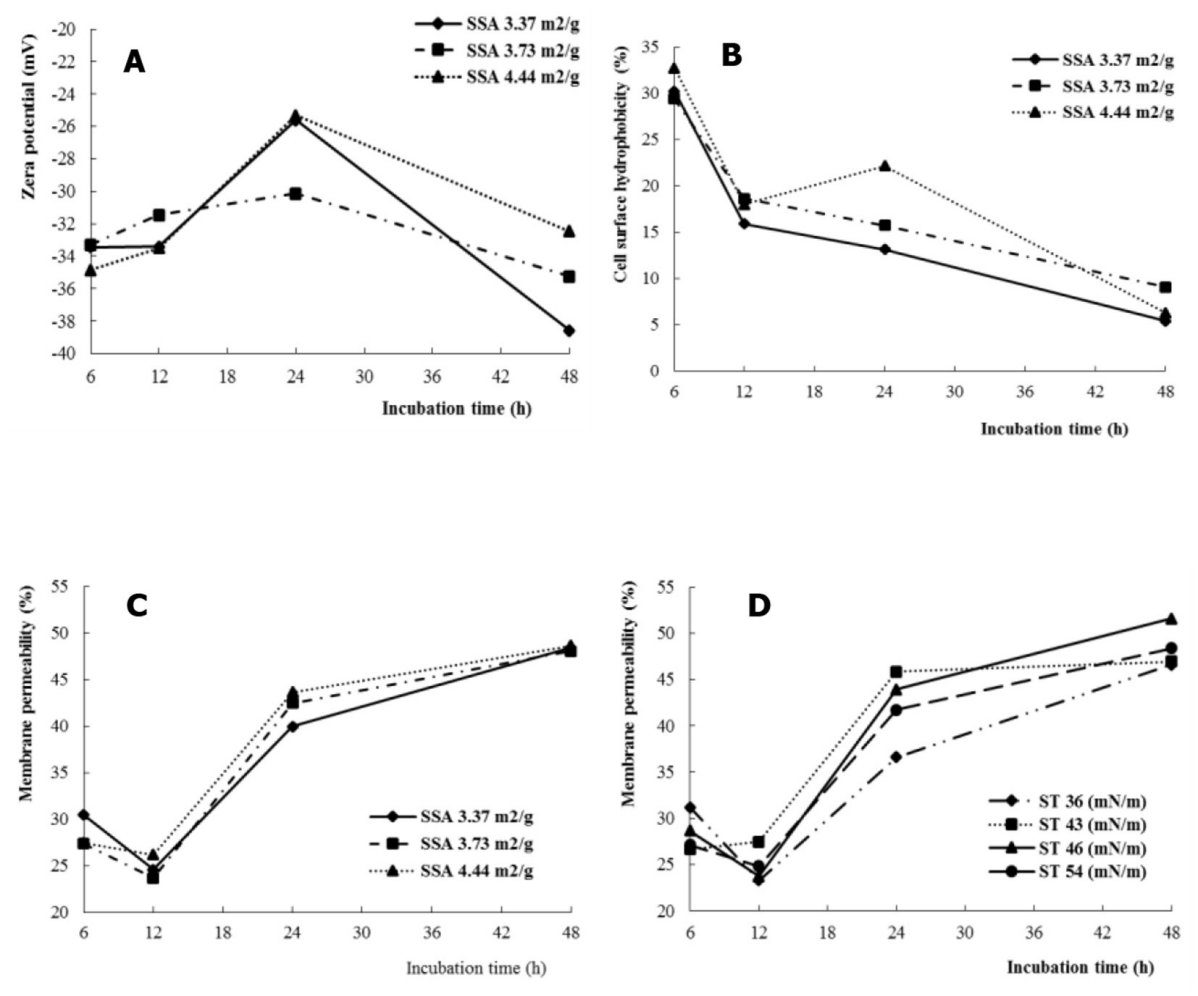
The SSA or ST interacted with fermentation time on cell membrane permeability of rumen microbes, Zeta potential **(A)**, cell surface hydrophobicity **(B)**, and membrane permeability **(C, D)**.

Zeta potential (ξ) changed with increasing incubation time (*P* < 0.01), and the effects of incubation time on ξ depended on the SSA of the substrate (SSA × time interaction, *P* < 0.05) (Table 2). At SSA = 3.37 m^2^/g, the increasing and decreasing magnitude of ξ from 12 to 24 h and 24 to 48 h, respectively, was greater than that of SSA = 3.73 m^2^/g (Figure 2A).

**Table 2:** Combinatorial interactions of SSA, ST, and incubation time on the physicochemical properties of rumen microbes *in vitro*.

**Item^1^**	**Physicochemical properties of ruminal microbiota** ^**2**^		
	**Zeta potential (**ξ**, mV)**	**Hydrophobicity (%)**	**Permeability (%)**
**SSA (m** ^2^ **/g)**			
3.37	−32.8	16.2^b^	35.8
3.73	−32.5	18.2^a^	35.4
4.44	−31.5	19.8^a^	36.5
SEM^a^	0.73	0.74	0.46
**ST (dynes/cm)**			
36	−33.3	18.6	34.4^b^
43	−33.3	19.1	36.7^a^
46	−30.3	18.1	37.0^a^
54	−32.2	16.3	35.5^ab^
SEM^b^	0.89	0.85	0.55
**Significance of effects**			
SSA	NS	^**^	NS
ST	NS	NS	^**^
SSA ^*^ ST	NS	NS	NS
Time	^***^	^***^	^***^
SSA ^*^ time	^*^	^*^	^*^
ST ^*^ time	NS	NS	^***^
SSA ^*^ ST ^*^ time	NS	NS	NS

^1^SSA, specific surface area; ST, surface tension; SEM^a^, standard error of least squares means for SSA; SEM^b^, standard error of least squares means for ST; SSA ^*^ ST, interaction between specific surface area and surface tension; Time, incubation time; SSA ^*^ time, interaction between specific surface area and incubation time; ST ^*^ time, interaction between surface tension and time; SSA ^*^ ST ^*^ time, interaction among SSA, ST and time.

^2^ ^a, b, c^Means with different superscripts differed (*P* < 0.05); ^*^, ^**^, and ^***^ mean *P* < 0.05, *P* < 0.01, and *P* < 0.001, respectively.

The hydrophobicity of the cell membrane of mixed rumen bacteria increased (*P* < 0.01) with increasing SSA of NDF. The effect of incubation time on hydrophobicity depended on the SSA of the substrate (SSA × time interaction, *P* < 0.05). The hydrophobicity of the cell surface of rumen bacteria decreased with increasing fermentation time (SSA = 3.37 and 3.73 m^2^/g), whereas it increased at 24 h (SSA = 4.44 m^2^/g) (Figure 2B).

Cell membrane permeability was higher (*P* < 0.01) for ST = 43 and 46 dynes/cm compared with ST = 36 dynes/cm. The effects of incubation time on cell membrane permeability were dependent on the SSA (SSA × time interactions, *P* < 0.05) and ST (ST × time interactions, *P* < 0.001). The increase in cell membrane permeability was greater for SSA = 4.44 m^2^/g (*P* < 0.05) than for SSA = 3.37 m^2^/g when the incubation time increased from 12 to 24 hours (Figure 2C). For ST = 36 dynes/cm, cell membrane permeability decreased with incubation time from 6 to 12 h, whereas it increased numerically for ST = 43 dynes/cm, and increased (*P* < 0.01) sharply with increasing fermentation time from 12 to 24 h than for ST = 36 dynes/cm (Figure 2D).

### Linear correlation analysis

**Figure 3:**
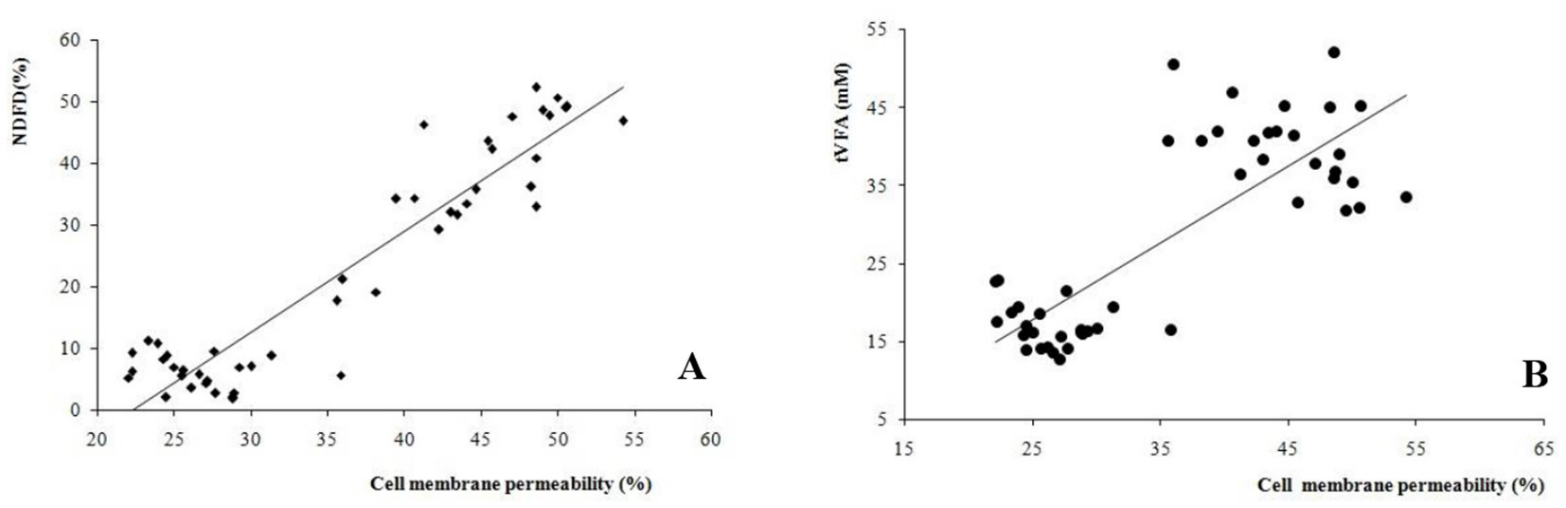
Correlations between bacteria cell membrane permeability and **(A)** neutral detergent fiber digestibility (NDFD, *r* = 0.937, *P* < 0.001), and **(B)** total volatile fatty acids (tVFA, *r* = 0.809, *P* < 0.001) among all the treatments and incubation times.

The correlation between cell membrane permeability of the microbiota was calculated, and the important fermentation value indices, NDFD, and tVFA (Figure 3). A high correlation (*r* = 0.937, *P* < 0.001) was found between permeability and NDFD. In addition, a high correlation (*r* = 0.809, *P* < 0.001) was established between microbiota permeability and tVFA concentration between all treatments and incubation times.

## Discussion

Volatile fatty acids (VFAs) are not only the primary end-products of carbohydrate metabolism in the rumen but also play critical roles in host energy metabolism and microbial homeostasis (33). The observed increase in tVFA with reduced surface tension suggests that the physicochemical properties of the fermentation medium can significantly influence microbial metabolic efficiency (17, 19). Reduced ST with nonionic surfactants or biosurfactants may enhance microbial interaction with substrates, promoting enzymatic action (34, 35). This finding aligns with studies demonstrating that microbial surfactants lower interfacial tension to improve fermentation efficiency (22, 36).

### Mechanistic insights into membrane permeability

The increased cell membrane permeability observed with lower ST values is consistent with findings that nonionic surfactants enhance microbial membrane fluidity, facilitating metabolite exchange and enzymatic leakage (15, 37). This mechanism allows extracellular enzymes to act more effectively on substrates, supporting the breakdown of complex carbohydrates (38, 39). The extracellular enzymatic activity of laccase, lignin peroxidase, and manganese peroxidase of *Phanerochaete chrysosporium* was increased with increasing permeability under the electronic field (30). Gram-negative bacteria are surrounded by an outer membrane consisting of a complex arrangement of glycerolphospholipids (GPL) (40). Their main function is to serve as a permeability barrier to allow the influx of essential nutrients while excluding harmful compounds, such as antibiotics and antimicrobial peptides (41, 42). There are strong positive correlations between cell membrane permeability and NDFD or tVFA. The high linear co-relationship between cell membrane permeability and fermentation parameters, such as NDFD (*r* = 0.937) and tVFA production (*r* = 0.809), underscore the critical role of permeability in microbial fermentation dynamics (15). Theoretical models suggest that optimal membrane permeability supports both nutrient uptake and the secretion of secondary metabolites, such as VFAs. This dual role highlights the centrality of permeability in microbial ecosystem stability and efficiency (43, 44). In general, the functions of signal transduction, secretion of active substances, nutrient absorption, metabolite excretion, and biological membrane function of the microbiota are influenced by cell membrane permeability (45), which in turn is related to the zeta potential and hydrophobicity properties of the membrane (46).

### Role of specific surface area and surface tension in hydrophobicity and adhesion

SSA significantly influenced microbial hydrophobicity, which is essential for microbial adhesion to feed particles and biofilm formation. Increased SSA provides more adsorption sites, enhancing microbial colonization and fiber degradation efficiency. These results are consistent with earlier findings showing that hydrophobicity promotes microbial adhesion, an essential precursor to effective fermentation.

Cell adhesion of microbes to the substrate, which is a prerequisite for bacterial colonization and proliferation, is influenced by cell surface hydrophobicity (47, 48). Higher hydrophobic strains have stronger adhesion ability (49, 50). We reported that hydrophobicity increased with increasing SSA, and higher hydrophobicity occurred in the first fermentation phage. We hypothesize that higher SSA increases the activity and adhesiveness of the microbiota in the rumen by altering its surface hydrophobicity. Interestingly, while SSA had a pronounced effect on microbial hydrophobicity, ST did not significantly alter this property. This observation underscores the dominant role of substrate characteristics in influencing microbial adhesion, emphasizing the importance of feed preparation in optimizing ruminal fermentation. Surface hydrophobicity was not affected by the ST of the medium. The lower hydrophobicity of the cell surface was evident during the incubation period. In previous studies, rumen microbes' hydrophobicity was related to bacterial aging, and a high value was found in freshly isolated strains of *Staphylococcus aureus* or *Serratia* spp. (51, 52). Given the complexity of the rumen microecosystem, further studies are needed to explain the relationships between the surface properties of the rumen microbiota and the adhesion process, microbial proliferation, and microbial enzyme secretion.

### Zeta potential and microbial stability

Zeta potential (ξ), a measure of surface charge, reflects the electrostatic interactions governing microbial and dispersion. In this study, ξ remained unaffected by ST or SSA, except for temporal changes. The reduction in ξ after 24 h may result from microbial aggregation and the accumulation of dead cells, which alter surface charge distribution (53). Although ξ showed a minimal direct correlation with membrane permeability, its role in maintaining microbial dispersion and biofilm integrity is essential (54, 55). In addition, the surface charge of bacteria is also influenced by the growth medium, bacterial phages, and bacterial surface structure (56). Membrane permeability can be directly affected by membrane zeta potential through a class of special channels known as voltage-gated ion channels or voltage-dependent ion channels, such as sodium/calcium/channel proteins (α-helical transmembrane segments, S1-S6) with a particular ion selectivity and voltage dependence (57, 58). The cell surface of the rumen microbiota was negatively charged, referred to as a net negative charge (56). The negative charges of the membranes increased with increasing fermentation time up to 24 h and decreased the resistance of the microbe attached to the substrate (59, 60). This indicates that the main adhesion of rumen microbes to the substrate occurred before 24 h.

### Interdependence of physicochemical properties

The interplay between hydrophobicity, membrane permeability, and zeta potential provides a comprehensive understanding of microbial responses to environmental changes (48). Manipulating SSA and ST can selectively enhance desirable microbial properties, such as adhesion (61, 62) and enzyme secretion while mitigating inhibitory factors like excessive aggregation (63, 64). These findings align with current research strategies to tailor fermentation environments for optimized microbial activity (10, 65).

### Practical implications for livestock nutrition and future directions

This study highlights actionable strategies for improving feed formulation in livestock nutrition. Increasing SSA through particle processing or additive application enhances microbial adhesion and fiber degradation, while moderate reductions in ST using bio-/nonionic- surfactants or other agents optimize enzymatic activity. These approaches can significantly enhance feed efficiency, reduce waste, and promote sustainable livestock production. Future studies should explore the molecular mechanisms underlying the observed effects using advanced omics technologies. Metagenomics and metabolomics can provide deeper insights into how SSA and ST influence microbial community composition and metabolic pathways, paving the way for precision management of ruminal fermentation.

## Conclusions

In the rumen, both the large specific surface area of the fiber and the low surface tension of the inoculum result in increased propionate production and decreased acetate-to-propionate ratios by disrupting the microbiome ecosystem. Cell surface properties of rumen microbes, including hydrophobicity and permeability, change with substrate properties and interfacial properties of the medium. In addition, neutral detergent fiber digestibility and total volatile fatty acid correlate strongly with the cell membrane permeability of the rumen microbiota. These results suggest that physical environmental properties may be critical in regulating the physical properties of the rumen microbiota and the balanced ecosystem.

## Data Availability

The raw data supporting the conclusions of this article will be made available by the authors, without undue reservation.
